# New Works

**Published:** 1845-06

**Authors:** 


### NEW WORKS.

Professor Gross is now in Philadelphia, superintending the publication of a new edition of his Elements of Pathological Anatomy, which will be issued in a single volume during the summer. The new edition will embrace whatever has been furnished to the author by his reading or observation in the last five years, not contained in the present edition, and will make an addition to the work of nearly three hundred pages. Many portions have been re-written, a number of new chapters have been introduced, and the illustrations by woodcuts multiplied to an extent which must very materially enhance the value of the treatise to students of medicine.

Professor Gross has also been engaged upon a new edition of Liston's Elements of Surgery, which will be ready for press early in the autumn. Y.

